# Recurrent vitreous occlusion of glaucoma drainage device tube in a patient with glaucoma in aphakia: a case report

**DOI:** 10.1186/1757-1626-3-55

**Published:** 2010-02-10

**Authors:** Ghee Soon Ang, Yi Wei Goh, Augusto Azuara-Blanco

**Affiliations:** 1 Department of Ophthalmology, Aberdeen Royal Infirmary, Foresterhill, Aberdeen AB25 2ZN, UK

## Abstract

Patients with spontaneous lens dislocation and glaucoma can be challenging to manage. We present a forty-six year old Caucasian lady who was referred with bilateral high intraocular pressure, and was subsequently diagnosed with glaucoma in association with lens dislocation and Marfan syndrome. Baerveldt glaucoma drainage device tubes were inserted in both eyes due to poor response to medical therapy. However, this was complicated by recurrent vitreous occlusion of both glaucoma drainage tubes requiring further multiple surgical interventions. There have not been any further recurrences of vitreous incarceration or posterior segment complications since, but the patient remains under close follow-up.

## Case presentation

A 46-year old Caucasian female was originally referred to the Eye Casualty department by the community optometrist because of bilateral raised intraocular pressures (IOPs) in the thirties. At Eye Casualty, she was additionally noted to be aphakic. She was started on topical Cosopt bd to both eyes, and was referred on to the Glaucoma Clinic. Interestingly, she had not been informed by the optometrist that she was aphakic.

**Figure 1:**
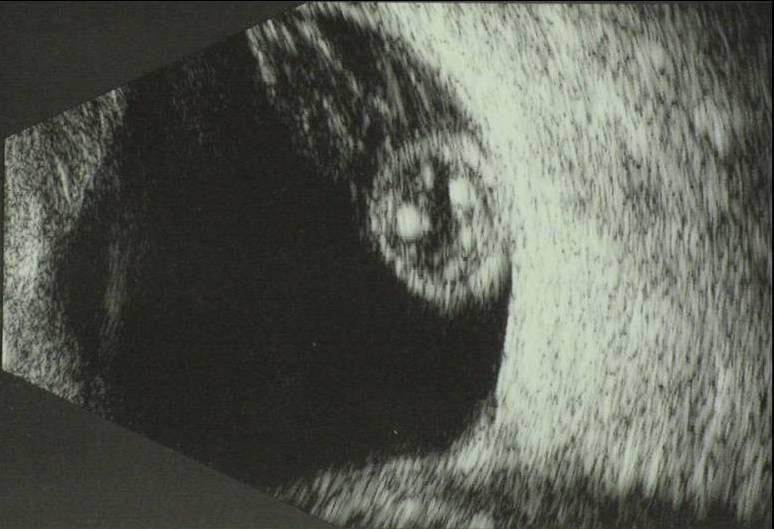
**Ocular ultrasound of the left eye demonstrating the dislocated lens in the vitreous cavity**. Ocular ultrasound of the left eye at presentation demonstrating the dislocated lens (heterogeneous hyperechogenic oval-shaped mass) within the vitreous cavity (hypoechogenic).

At her first presentation to the Glaucoma Clinic in May 2007, her IOPs had reduced to 22 mmHg in the right and 20 mmHg in the left. Her best-corrected visual acuity (BCVA) was 6/12 N5 right and 6/36 N24 left. She was bilaterally aphakic. Gonioscopy showed open angles. The corneas were clear with no endothelial pigmentation, and the irides did not have any transillumination defects. The anterior chambers (AC) were deep and quiet, with no sign of any vitreous prolapse. The optic discs were both pale and cupped, with a cup-disc ratio of 0.8. Both lenses were dislocated into the vitreous cavity. The rest of the dilated fundal examination was normal; no vitritis or retinal breaks were detected. The dislocated lenses and absence of retinal breaks were confirmed on ocular ultrasound (Figure 1). Timolol caused palpitations, and was discontinued.

She was a non-smoker, was generally fit and well, and had 2 children. She had previous hysterectomy, mild asthma and osteoporosis, and was on hormone replacement therapy. Apart from left amblyopia, she had no other ocular history of note, including trauma or surgery. There was no family history of any inherited ocular conditions. Her father was a type 2 diabetic with an undiagnosed 'eye problem', and had died unexpectedly from a myocardial infarction in his forties.

In view of her apparent bilateral spontaneous lens dislocation, a general physical examination was performed. She was 170 cm tall, with an arm span to height ratio of more than 1.05. She also had a high arched palate and joint hypermobility. No heart murmurs were heard on auscultation. Echocardiography revealed a normal aortic arch, but a dilated ascending aorta and mild aortic regurgitation. Based on the ocular, skeletal and cardiac findings, the patient was diagnosed with Marfan syndrome. Subsequently, her son was also diagnosed with Marfan syndrome. They both remain under current review by the cardiologists.

Despite maximal topical medical therapy (latanoprost 0.004%, dorzolamide 2% and brimonidine 0.2%), her IOPs remained high at 29 mmHg right and 41 mmHg left. Additional IOP control was only achieved with oral acetazolamide. Seven months after her first presentation, the option for surgical treatment with a glaucoma drainage device (GDD) was brought up. The risks and benefits of surgery to her left eye were discussed at length. In addition to the ocular risks, the anaesthetic risks associated with Marfan syndrome were also discussed. She agreed, and was listed for left Baerveldt implant insertion under general anaesthetic.

The 350 mm^2 ^Baerveldt device implantation at the superotemporal quadrant of the left eye proceeded routinely without any complications. As vitreous was not present in the AC nor was it at the pupillary plane, anterior vitrectomy was not performed at the same setting. Two weeks postoperatively, her IOPs were 42 mmHg right and 39 mmHg left. Her left pupil was peaked, with vitreous plugging the tube opening. Vitreolysis was successfully performed with Nd:YAG laser. Unfortunately, 2 weeks later, vitreous had occluded the left Baerveldt tube again. Laser vitreolysis was repeated successfully. A vitreo-retinal consultation was sought, and it was felt that the risk of retinal detachment from pars plana vitrectomy (PPV) outweighed the benefits, especially when the incarceration had been successfully treated with laser vitreolysis.

Unfortunately, she developed vitreous incarceration for the third time. By then both IOPs had become uncontrollable (34 mmHg right and 31 mmHg left) despite maximal topical and systemic medical therapy. It was decided that surgery was required for both eyes - GDD for the right, and anterior vitrectomy for the left. Two weeks later, a 350 mm^2 ^Baerveldt implant was placed in her right eye at the superotemporal quadrant, without any complications. Again, no vitreous was detected in the AC intraoperatively, and anterior vitrectomy was therefore not performed. For the left eye, anterior vitrectomy was performed with a 25-gauge vitrectomy probe via a corneal approach. At the end of surgery, no vitreous could be seen in the AC, and flushing of the Baerveldt tube resulted in an increase in the height of the conjunctival bleb.

One month postoperatively, vitreous had plugged the left tube for the fourth time. A second more extensive anterior vitrectomy was therefore performed with an AC maintainer and a 25-gauge vitrectomy probe via the pars plana. The same evening after her second left anterior vitrectomy, she became symptomatic with right eye pain and nausea. Her right IOP had increased to 70 mmHg with associated with vitreous plugging of the tube. The next day, she underwent right anterior vitrectomy with a pars plana approach. Unfortunately, six weeks later, vitreous was found to be plugging the right tube for the second time, necessitating a second right pars plana anterior vitrectomy.

**Figure 2:**
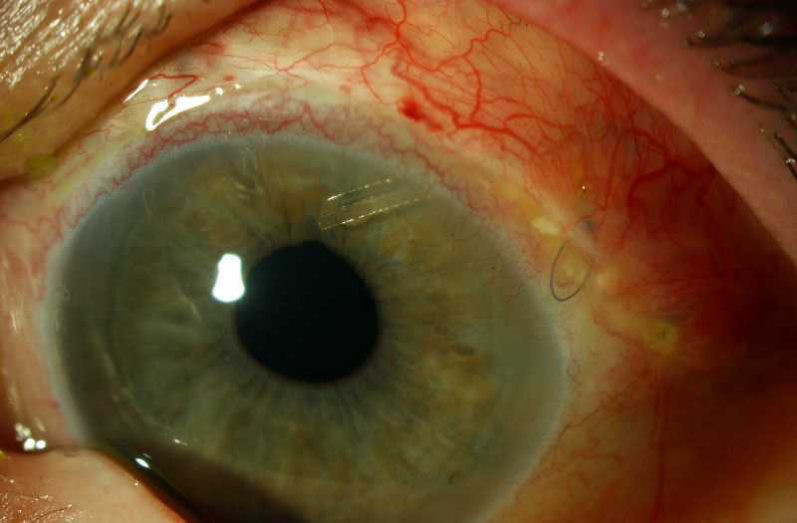
**Colour photograph of the left eye showing the Baerveldt implant tube with a round pupil**. Colour photograph of the left eye one week following her second anterior vitrectomy (via the pars plana) - the Baerveldt implant tube looked patent, the pupil was round and there was no vitreous in the anterior chamber.

Despite this, her right pupil became peaked 10 days later with an IOP of 22 mmHg, although no obvious vitreous strands could be seen in the AC. A repeat vitreo-retinal consultation was sought, and she subsequently underwent right PPV and heavy liquid-assisted lens removal via a scleral tunnel section. At the last clinic visit (10 weeks after her PPV), her IOPs were 11 mmHg right (with brimonidine 0.2% bd and dorzolamide 2% bd) and 15 mmHg left (with brimonidine 0.2% bd, dorzolamide 2% bd and travoprost 0.004% nocte). So far, there have been no recurrences of vitreous incarceration to either tube (Figure 2), and no posterior segment complications have occurred.

## Discussion

Marfan syndrome is an inherited mixed connective tissue disorder which usually presents as a constellation of signs and symptoms involving 2 or more organ systems such as skeletal, joints, dermatological, dural sac, lungs, ocular or cardiac malformations. It is an autosomal dominant condition, and is due to a mutation in the fibrillin-1 gene located on chromosome 15q21. It is the commonest inherited multi-system disorder of connective tissue. Approximately 80% of Marfan syndrome cases are inherited, while the remainder are due to de novo mutations. Despite advancements in genetic testing, the diagnosis of Marfan syndrome is still dependent on clinical features (Table 1), as based on the modified Ghent criteria listed below:[1]

**Table 1:** Major Modified Ghent criteria for Marfan syndrome

System	Major features	Minor features	Requirement
*Skeletal*	Pectus carinatum Pectus excavatum needing surgery Arm span to height ratio >1.05 Wrist (Walker) and thumb (Steinberg) signs Scoliosis >20° or spondylolisthesis Reduced elbow extension <170° Pes planus Protrusio acetabuli	Pectus excavatum Thoracic lordosis Scoliosis <20° Joint hypermobility Highly arched palate Dental crowding Typical facies (dolichocephaly, malar hypoplasia, enophthalmos, retrognathia, downward slanting palpebral fissures)	*2 major features;* *Or, 1 major and 2 minor features*
*Ocular*	Ectopia lentis	Flat cornea Axial length >23.5 mm Hypoplastic iris or ciliary muscle causing reduced miosis Nuclear sclerosis under the age of 50 Glaucoma under the age of 50 Retinal detachment	*Major feature;* *Or, 2 minor features*
*Cardiovascular*	Dilatation of the ascending aorta involving the sinuses of Valsalva Dissection of the ascending aorta	Mitral valve prolapse Dilatation of the main pulmonary artery under the age of 40 Calcification of the mitral annulus under the age of 40 Dilatation or dissection of the thoracic or abdominal aorta under the age of 50	*1 minor feature*
*Pulmonary*	None	Spontaneous pneumothorax Apical blebs	*1 minor feature*
*Skin*	None	Striae atrophicae Recurrent or incisional hernia	*1 minor feature*
*Dura*	Dural ectasia - enlargement of the dural sac in the lumbosacral region (CT or MRI)		*Major feature*

1. A first-degree relative with confirmed Marfan syndrome, major criteria in one organ system, and involvement of a second organ system; or

2. A fibrillin-1 gene mutation known to cause Marfan syndrome, major criteria in one organ system, and involvement of a second organ system; or

3. Major criteria in two organ systems, and involvement of a third organ system

Our case report illustrates the difficulties and challenges that can arise when managing a Marfan syndrome patient with lens dislocation and glaucoma. Firstly, it was difficult to ascertain the mechanism or mechanisms of glaucoma. Secondly, glaucoma in aphakia can be very difficult to control, and this was certainly the case for our patient. Thirdly, lens dislocation and aphakia are significant risk factors for retinal detachment in Marfan syndrome. Any surgical procedure involving or affecting the vitreous will considerably increase this risk.

There is a general consensus that trabeculectomy in aphakia would be less successful than in phakic eyes [2,3]. There have been 2 randomised trials comparing MMC-augmented trabeculectomy with GDDs (one with Ahmed glaucoma implants, and the other with 350 mm^2 ^Baerveldt implants) [4,5], both demonstrating no difference in IOP control at one year. However, these 2 trials did not include glaucoma patients with aphakia. We eventually proceeded with 350 mm^2 ^Baerveldt device implantation as our primary surgical procedure on the basis that trabeculectomy would have a higher rate of failure because of our patient's aphakic status and probable tendency towards trabeculectomy bleb scarring due to her relatively young age. We acknowledge that GDD implantation is not without its complications, which include hypotony, endophthalmitis, tube exposure, and ocular motility problems.

It may be possible that combining the Baerveldt implant surgery with adequate core anterior vitrectomy or PPV during the primary surgery for our patient would have reduced the risk of vitreous incarceration into the tube. Desatnik *et al *reported that vitreous incarceration in implant tubes occurred despite 6 of the series of 8 eyes having previous anterior vitrectomy, and concluded that anterior vitrectomy was insufficient in preventing vitreous occlusion [6], However, in these eyes, the implant was either inserted into the vitreous cavity, or the vitreous was already present in the AC during implant surgery (i.e. insufficient anterior vitrectomy). Several retrospective studies have found combined PPV with GDD implantation to be successful in controlling IOP in refractory glaucoma, including patients with aphakic glaucoma [7-9]. However, this increases the risk of posterior segment complications, such as retinal detachment, epiretinal membrane, and cystoid macular oedema. We did not perform PPV during the primary procedure because at that time, it was felt that it would have significantly increased the risk of retinal detachment, which may in turn be complicated by severe proliferative vitreoretinopathy or giant retinal tear with consequent poor visual outcome. This was particularly important for her non-amblyopic eye.

Pars plana vitrectomy, if performed properly, would ensure that vitreous would not become incarcerated in the Baerveldt tube opening, and would also remove a potential cause for intraocular inflammation and phacolytic glaucoma. In a series of 8 eyes of 8 patients with vitreous incarceration in the Baerveldt implant, 6 were successfully treated with PPV and 1 with Nd:YAG vitreolysis alone [6]. Cyclodiode laser ciliary ablation is also another option, with a moderate success rate (48%) in glaucoma in aphakia [10]. However, with the considerable potential complications of hypotony, phthisis bulbi, retinal detachment, and macular oedema, it will only be realistically considered as a last resort for our patient, or if no useful visual function remains. Our patient eventually required multiple anterior vitrectomies to both eyes and full PPV to the right. Looking back in retrospect, it could be argued that PPV, or at least anterior vitrectomy, should also have been performed during the primary surgery.

## Conclusion

This report illustrates the difficulties in balancing potential benefits against potential risks of surgical interventions, especially for the non-amblyopic eye. A full PPV for our patient could potentially result in devastating posterior segment complications, causing our patient to be significantly worse off compared to before surgery. It is also vital to remember that Marfan syndrome is a systemic condition, where aortic dissection is an important but preventable cause of mortality. The importance of a general medical and systemic examination in a patient with apparent spontaneous lens dislocation to detect any associated systemic disorder cannot be emphasised enough.

## Abbreviations

GDD: glaucoma drainage device; IOP: intraocular pressure; BCVA: best corrected visual acuity; AC: anterior chamber; PPV: pars plana vitrectomy.

## Consent

Written informed consent was obtained from the patient for publication of this case report and accompanying images. A copy of the written consent is available for review by the Editor-in-Chief of this journal.

## Competing interests

The authors declare that they have no competing interests.

## Authors' contributions

GSA, YWG and AAB were major contributors in writing the manuscript. All authors read and approved the final manuscript.

## References

[B1] De Paepe A Devereux RB Dietz HC Hennekam RC Pyeritz RE Revised diagnostic criteria for the Marfan syndrome Am J Med Genet 1996 62 417 426 10.1002/(SICI)1096-8628(19960424)62:4<417::AID-AJMG15>3.0.CO;2-R 8723076

[B2] Heuer DK Gressel MG Parrish RK Anderson DR Hodapp E Palmberg PF Trabeculectomy in aphakic eyes Ophthalmology 1984 91 1045 1051 6493713 10.1016/s0161-6420(84)34196-3

[B3] Tomey KF Traverso CE The glaucomas in aphakia and pseudophakia Surv Ophthalmol 1991 36 79 112 10.1016/0039-6257(91)90124-X 1957248

[B4] Wilson MR Mendis U Smith SD Paliwal A Ahmed glaucoma valve implant vs trabeculectomy in the surgical treatment of glaucoma: a randomized clinical trial Am J Ophthalmol 2000 130 267 273 10.1016/S0002-9394(00)00473-6 11020403

[B5] Gedde SJ Schiffman JC Feuer WJ Herndon LW Brandt JD Budenz DL Treatment outcomes in the tube versus trabeculectomy study after one year of follow-up Am J Ophthalmol 2007 143 9 22 10.1016/j.ajo.2006.07.020 17083910

[B6] Desatnik HR Foster RE Rockwood EJ Baerveldt G Meyers SM Lewis H Management of glaucoma implants occluded by vitreous incarceration J Glaucoma 2000 9 311 316 10958604 10.1097/00061198-200008000-00005

[B7] Scott IU Alexandrakis G Flynn HW Jr Smiddy WE Murray TG Schiffman J Gedde SJ Budenz DL Fantes F Parrish RK Combined pars plana vitrectomy and glaucoma drainage implant placement for refractory glaucoma Am J Ophthalmol 2000 129 334 341 10.1016/S0002-9394(99)00363-3 10704549

[B8] Varma R Heuer DK Lundy DC Baerveldt G Lee PP Minckler DS Pars plana Baerveldt tube insertion with vitrectomy in glaucomas associated with pseudophakia and aphakia Am J Ophthalmol 1995 119 401 407 7709964 10.1016/s0002-9394(14)71224-3

[B9] Gandham SB Costa VP Katz LJ Wilson RP Sivalingam A Belmont J Smith M Aqueous tube-shunt implantation and pars plana vitrectomy in eyes with refractory glaucoma Am J Ophthalmol 1993 116 189 195 8352304 10.1016/s0002-9394(14)71284-x

[B10] Schlote T Grub M Kynigopoulos M Long-term results after transscleral diode laser cyclophotocoagulation in refractory posttraumatic glaucoma and glaucoma in aphakia Graefes Arch Clin Exp Ophthalmol 2008 246 405 410 10.1007/s00417-007-0708-0 17965873

